# Narratives About COVID Challenges in Mexican American Couples as Predictors of Relationship Satisfaction

**DOI:** 10.1111/famp.70176

**Published:** 2026-07-02

**Authors:** Hollen N. Reischer, Hayley C. Fivecoat, Lena Blum, Chrishane N. Cunningham, Stacey Ho, Erika Lawrence

**Affiliations:** ^1^ Department of Psychology University at Buffalo, State University of New York Buffalo New York USA; ^2^ Center for Applied Psychological and Family Studies The Family Institute at Northwestern University Evanston Illinois USA; ^3^ Department of Psychology Northwestern University Evanston Illinois USA; ^4^ Department of Counseling Psychology University of Denver Denver Colorado USA; ^5^ Department of Psychology and Educational Services Fordham University New York City New York USA

**Keywords:** APIM, couples, COVID‐19, Hispanic, Mexican American, narrative identity, romantic relationships

## Abstract

The objective of this study was to describe the narrative themes that emerged in Mexican American couples' stories about their relationship challenges at the beginning of the COVID‐19 pandemic—during lockdown—and to examine how those themes related to their relationship satisfaction. We surveyed 58 male–female married couples (*n* = 116 participants; 73.33% Mexican American) recruited from the Southwest United States and enrolled in a longitudinal study of relationship functioning. Spouses wrote stories about the greatest COVID‐19–related challenges they were facing as a couple. To explore associations between relationship satisfaction and the lived experience of COVID‐19, stories were coded for established narrative identity themes (agency, communion, affect, meaning‐making) and themes developed to account for the context of marital challenges (challenge outcomes, couple stress responses, familismo, overriding positive regard). Relationship satisfaction was associated with six narrative themes for wives (0.29 ≤ |*r*| ≤ 0.56) and three themes for husbands (0.29 ≤ |*r*| ≤ 0.40). Actor–partner interdependence modeling showed six actor effects and one partner effect for wives but no significant effects for husbands. This research provides novel insight into the challenges faced by Mexican American couples during the pandemic and suggests great promise for using narrative identity research to understand how individuals' narrations of couple stories are linked to relationship satisfaction. Further, our findings suggest that the narrative themes characterizing how conflict is storied by partners within the couple therapy room might be directly targeted as a means to improve relationship satisfaction.

## 1 Introduction

Relationship science has increasingly included diverse populations in recent years, yet the preponderance of knowledge about couple experiences and applications to clinical practice remains primarily informed by studies of White, non‐Hispanic adults (Orengo‐Aguayo 2015). However, changes in the US population over the past decades highlight the urgency of expanding this research. By 2023, the Hispanic^1^ population comprised approximately 20% of the US population, and this proportion is expected to continue growing (US Census Bureau 2024). Hispanic adults face unique challenges relative to non‐Hispanic adults in the United States, including hostile immigration policies, ethnic discrimination, and economic disparities such as lower household incomes and greater difficulty paying for basic needs (Ogan et al. 2024; Scherer and Mayol‐García 2022). The largest subgroup within the US Hispanic population is Mexican Americans, or Hispanics of Mexican origin who trace their ancestry to Mexico or have immigrated from Mexico (Peña et al. 2023).

Unfortunately, research on couples in which one or both partners are Mexican American is scarce, despite the size of this population and the fact that disparities and inequities experienced by this group are associated with poorer individual and relationship functioning (Helms et al. 2014; Ogan et al. 2024; Parke et al. 2004). Additionally, Mexican Americans must often navigate culturally specific relationship complexities, balancing traditional cultural values such as close family ties and respect for older generations with the demands of modern American society (Chen et al. 2021; Gonzalez‐Detrés et al. 2025). These intersecting identities can shape approaches to and experiences of parenting, work, and relationship dynamics in distinct ways (Archuleta 2015; Orengo‐Aguayo 2015).

Such challenges become even more pronounced during periods of severe chronic stress, such as the COVID‐19 pandemic. Indeed, at the outset of the pandemic, 57% of Hispanic individuals were designated as “essential workers” (US Bureau of Labor Statistics 2019) and as such were at increased risk of exposure to COVID‐19. The higher likelihood of Latinx households to be multigenerational added stress about increased health risks to older and other vulnerable household members (Gould et al. 2020). During the pandemic, Mexican American mothers reported high levels of anticipatory stigma, fear that a family member would be deported, and fear that they would be unable to care for their children, all of which were linked to poorer mental health (Non et al. 2025). Poorer mental health was also observed among those Mexican American individuals who experienced pandemic‐related declines in the quality of their family relationships (Volpert‐Esmond et al. 2023).

The present study adds to the sparse literature on Mexican American couples more broadly and to the literature on their navigation of prolonged, extreme stressors. The purpose of the study was to elucidate how Mexican American partners narrated their experiences of relationship challenges during one of the most stressful periods in recent history—lockdown at the beginning of the COVID‐19 pandemic—and to assess how themes in those narratives related to their relationship satisfaction. We employed a narrative identity approach, which was well‐suited to our goal of obtaining and interpreting accounts of how spouses navigate relationship challenges. In the next section, we introduce this methodology and describe its application to relationship science.

### 1.1 Using a Narrative Identity Approach to Elucidate Couple Experiences

The narrative identity approach examines how people narrate their life experiences to themselves and others, with a focus on how people make meaning of these experiences and integrate these meanings into their ideas about who they are and why they do what they do (McAdams and McLean 2013). Narrative identity research methods typically entail eliciting stories of specific life events and prompting individuals to explain how their stories reflect aspects of their identity. These methods have been shown to offer incremental validity in predicting well‐being over and above other personality measures (Adler et al. 2015) and to be relevant to a broad array of psychological outcomes (McLean et al. 2020).

Narrative themes are features that characterize the affective, motivational, and meaning‐making qualities of narratives—for example, describing the predominant emotional tone or the depth of a narrator's integration of an event into their broader story. Many narrative themes have been translated into quantitative variables that may be measured to describe individual differences in how stories are narrated (McLean et al. 2020). Previous research has shown that narrative themes predict self‐report measures of individual well‐being, with particularly strong evidence for the association between well‐being and narratives rich with motivational themes, such as agency and communion (Thomsen et al. 2025; Ture et al. 2026). In stories about adversity and negative life events, narrative themes of accommodation to challenges, emotional closure, and happy endings have been found to predict better psychological functioning (Adler and Poulin 2009; King et al. 2000; Mansfield et al. 2010).

Narrative identity methods have rarely been used in couples research, though early investigations suggest the method is similarly powerful in a dyadic context (Bühler and Dunlop 2019; Dunlop et al. 2020; Panattoni and Thomsen 2018; Skerrett 2010). Emerging research exploring links between narrative themes in stories that romantic partners tell about one another and relationship‐relevant outcomes suggests that higher relationship satisfaction may be predicted by narratives with more positive affective tone (Dunlop et al. 2020; Frost 2012) and more communal language and themes (Agnew et al. 1998; Fitzsimons and Kay 2004; Frost 2012). Whereas prior studies have elicited participants' narratives either about their love life stories (e.g., “What has been the high point of your love life?”) or their own open‐ended life stories (e.g., “What has been the turning point of your life?”), few studies have examined narrative identity themes in participants' accounts of coping with a major stressor, and how these relate to relationship satisfaction.

The present study is among the first to apply a narrative identity approach in relationship science, and we chose to use this approach for several reasons. First, we expected the narrative identity method to yield novel, nuanced, and contextual information about how couples navigate times of severe chronic stress in such a way that we could better capture the complexity of this lived experience unfolding across multiple levels of the socioecological system (e.g., individual, partner, family, community, society). In particular, this methodology allowed us to capture qualities of the stories that partners hold of their relationships during times of stress. Second, Mexican Americans are often the objects, rather than the subjects or agents, of cultural narratives; we see the narrative identity approach as providing one way to enable study participants to experience greater agency in the research process. Third, our sample was recruited from an ongoing longitudinal study of couples with whom the study staff had already developed trust and rapport through several waves of data collection, including in‐depth live interviews. Having such pre‐existing relationships with dyads offered a unique opportunity to more readily obtain perspectives from both spouses in a couple. Overall, we chose to employ a narrative identity approach—in this instance, collecting written narrative responses in addition to self‐report measures—to gather richer data than standardized measures alone could provide. We paired this with actor‐partner interdependence modeling to fully utilize the dyadic nature of these data (Cook and Kenny 2005), which we expected to enrich our contribution to relationship science and to the narrative identity literature.

### 1.2 Purpose of the Present Study

The purpose of the present study was to clarify the narrative themes that emerge in Mexican American couples' stories of relationship challenges during the beginning of the COVID‐19 pandemic and to examine how those themes relate to individuals' and partners' relationship satisfaction. We included narrative themes representing the individual narrator's qualities and those representing the narrator's assessment and expression of relational qualities. Themes measuring individual characteristics were drawn from the extant narrative identity literature: agency (the ability to affect one's own life, initiate change, and achieve some degree of control over one's experiences), communion (interpersonal connection, intimacy, love, belonging, union, friendship, and caring), affect (emotional tone of the narrative), and meaning‐making (reflecting on and learning something new from an event). Four narrative themes capturing relational characteristics were developed after reviewing relevant research from the qualitative relationship narrative literature, as detailed further in our Method: challenge outcome (extent to which the challenge or problem is described as resolved), couple stress response (degree of relationship cohesion or closeness versus more conflict or disconnection resulting from the challenge), familismo (connectedness to non‐spouse family members or endorsement of familial support beliefs), and overriding positive regard (tendency to downplay or contextualize challenges by linking them to global, abstract positive feelings about the relationship or partner).

We hypothesized that the two individual themes of higher affect and communion, and the four relationship themes (challenge outcome, couple stress response, familismo, and overriding positive regard), would be positively and significantly associated with relationship satisfaction. We did not generate a priori hypotheses related to agency or meaning‐making, as these have not previously been empirically or theoretically linked to relationship satisfaction; however, because these themes were coded and examined for another purpose (see Reischer et al. 2026), we chose to include them for transparency, which is essential to good social science research (Miguel et al. 2014). Because of the scarcity of literature to guide hypotheses on partner effects in the narrative identity literature, we did not make hypotheses about them.

## 2 Method

Data were collected in May and June of 2020 in the Southwest of the United States. During this time, the state's COVID‐19 case numbers doubled, and a statewide stay‐at‐home order was mandated. After ending the lockdown, lawmakers mandated public closures and mask requirements. At the same time, immigration from Mexico was a topic of increasing national political discourse, and this sample was recruited from the Southwest close to the border. In the present study, couples were not asked about their immigration status or citizenship due to the sensitive nature of those questions. Nevertheless, it seems likely that these families faced immigration‐related stressors in addition to pandemic‐related stressors.

### 2.1 Participants

Participants were recruited from an ongoing longitudinal study examining marital functioning over the first 6 years of marriage (Fivecoat et al. 2024). In that study, couples were recruited through marriage licenses. To be eligible for the study, at least one partner had to identify as Mexican American. Additionally, they had to be in their first marriage, have been married for 3–9 months, and be able to read and write in English. During the eligibility screening process, 73.33% of the enrolled participants identified as Mexican American, including 28.15% who identified as Multiracial (Mexican American and at least one other race). The remaining participants identified as non‐Hispanic White (24.44%), Asian American (1.48%), and Native American/Pacific Islander (0.74%). Demographics were collected at the baseline assessment, at approximately 6 months of marriage. At that time, individuals were 21–51 years old (*M* = 31.26, SD = 5.70). Incomes ranged from the lowest income category ($0–$9999) to the highest ($100,000+), with a median of $50,000–$59,000. Half (51.85%) of participants had a bachelor's degree or a higher level of education, 31.85% had an associate degree or some college, 13.33% had a high school diploma or GED, and 2.96% had some high school education.

All participants in the ongoing longitudinal study were invited to complete an online survey about their experiences during the pandemic. Responders (*n* = 146) had slightly higher personal income than nonresponders ($30,000–$39,000 and $20,000–$29,000, respectively) at baseline, but did not differ by gender, ethnicity, age, or employment status. Responders were included in the present study if both they and their spouse completed the survey, including the narrative questions (*n* = 116).

At the time of the present survey, approximately 4 years into marriage, 57.76% of present study participants reported having children in the home, and 10.34% reported having other family members living with them. Most (81.03%) participants were employed, with 35.34% working outside of the home, 37.93% working from home, and 6.03% working in mixed or unspecified settings. A sizeable minority had experienced furlough (18.97%) or job loss (12.07%) during the pandemic, with 3.45% furloughed and 2.59 still laid off at the time of the survey Further, 43.96% reported increased relationship stress or conflict specifically due to the pandemic, and 37.07% reported that ongoing, pre‐existing stressors were amplified because of the pandemic.

### 2.2 Procedure

Participants enrolled in the parent study, a longitudinal study of marriage, were contacted by email weekly for 4 weeks and invited to participate in an optional additional survey related to their experiences during the pandemic. Individuals who participated completed a 30‐min online survey that included the measures used in the present study. Individuals were instructed to complete the survey independently of their spouse. Participants were paid $25 each. All participants provided informed consent online for this specific survey. The survey and procedures were approved by the Northwestern University Institutional Review Board.

### 2.3 Measure of Relationship Satisfaction

We measured relationship satisfaction using the Couples Satisfaction Index (CSI‐16; Funk and Rogge 2007). Developed using item‐response theory methodology, this scale consists of 16 items (e.g., “I have a warm and comfortable relationship with my partner,” and “Our relationship is strong”) rated on 6‐ or 7‐point Likert scales. Scores may range from 0 to 81, with higher scores indicating higher relationship satisfaction. Inter‐item reliability was excellent (*α* = 0.97) in this sample. Items were summed to calculate a total score, and scores were square‐root transformed to correct for negative skew.

### 2.4 Narrative Identity Measure and Coding Processes

#### 2.4.1 Narrative Prompt

At the end of the online survey, participants were asked to describe the greatest challenge they had faced as a couple during the pandemic. The instructions began:

We'd like you to tell us about your relationship, as it relates to the COVID‐19 crisis, in your own words. For all questions, please don't worry about spelling or grammar, but do try to write in sentences, as if you were telling a story. Be as detailed as possible.

Participants were then presented with a series of prompts: (1) Please identify what has been the biggest challenge you've faced as a couple during the COVID‐19 crisis. What is the challenge or problem? (2) How have you addressed or dealt with this challenge or problem? (3) Is there anything in your past as a couple that made it easier or harder for you to face this challenge? (4) What meaning do you make of this challenge or problem? (5) What does this event say about you as a partner? and (6) About your marriage?

#### 2.4.2 Individual Narrative Codes

Four themes were coded pertaining to individual constructs. These codes were selected from the established narrative identity literature because they have often been tested in association with measures of (individual) well‐being (Adler et al. 2016) or because prior narrative identity research exploring romantic relationships has considered these (Dunlop et al. 2020; Frost 2012). Agency describes the narrator's expression of the ability to affect their own lives, initiate change, and achieve some degree of control over their experiences (Adler 2012). Agency was scored ranging from −2 (*lowest agency*) to 2 (*highest agency*). Communion describes the narrator's use of themes of interpersonal connection, intimacy, love, belonging, union, friendship, and care (Adler et al. 2012). Communion was scored using a range of −2 (*lowest communion*) to 2 (*highest communion*). Affect captures the emotional tone of the narrative, ranging from very negative or pessimistic to very positive or optimistic (McLean et al. 2020), and was scored between −2 (*very negative affect*) and 2 (*very positive affect*). Meaning‐making is scored when the narrator describes reflecting on and learning something new from an event, with scores ranging from 0 (*no meaning made*) to 3 (*insight*; McLean and Pratt 2006).

#### 2.4.3 Relationship Narrative Codes

We used a hybrid inductive‐deductive approach to develop the relationship themes (Kaur et al. 2025; Proudfoot 2023). We first familiarized ourselves with a subset of participant narratives to understand the scope of the content. Next, we developed a list of terms to search the literature in the areas of narrative identity (within personality science), relationship science, couple therapy, and Mexican American studies. At weekly team meetings, we discussed findings, drafted and revised a working codebook, and made further updates as we coded small portions of the dataset. Challenge outcome indicates the extent to which the challenge or problem the narrator identified is described as resolved, ranging from “completely unresolved” to “resolved and improved the relationship.” Challenge outcome scores ranged from −2 (*completely unresolved*) to 2 (*completely resolved*). Couple stress response is a rating of the degree to which the narrator describes an increase in relationship cohesion or closeness versus no change or more conflict or disconnection with their partner as a result of the described challenge and is coded from −1 (*increased conflict or disconnection*) to 1 (*increased closeness or cohesion*). Present (vs. absent) overriding positive regard indicates that the narrator downplays or contextualizes challenges by linking them to global, abstract positive feelings about the relationship or partner; this theme was coded as 0 when absent and 1 when present. Finally, present (versus absent) familismo indicates that the narrator describes a connection to non‐spouse family members or endorses beliefs such as a duty to provide material support to family or expectations to receive such support. Familismo was also coded as 0 when absent and 1 when present.

#### 2.4.4 Narrative Coding

The six prompts were evaluated as a single narrative and coded for eight narrative themes using well‐validated narrative identity methods (Adler et al. 2017; McLean et al. 2020; Syed and Nelson 2015). A detailed description of the training and coding process can be found in Reischer et al. (2026). In brief, four novice coders, blind to participants' self‐report scores on any variable, were trained by an expert coder to consistently apply qualitative (for descriptive themes) and quantitative (for narrative themes) coding schemes to individual narratives. A pair of coders coded each descriptive and narrative theme, and final reliability as indicated by intraclass correlation coefficients (Gamer et al. 2019) or prevalence‐ and bias‐corrected kappa for binary variables (Stevenson and Sargeant 2021) was acceptable to good across narrative themes. Final inter‐rater reliability statistics were affect (ICC = 0.85), agency (ICC = 0.70), challenge outcome (ICC = 0.63), communion (ICC = 0.76), couple stress response (ICC = 0.82), meaning‐making (ICC = 0.84), familismo (PABAK = 0.66; 83.33% agreement), and overriding positive regard (PABAK = 0.54; 76.47% agreement). Following independent coding, coders met to discuss any discrepancies in ratings to reach a final consensus score. If coders could not reach consensus, the expert coder was brought in to discuss and offer their own perspective, and the three reached consensus together (Syed and Nelson 2015). Consensus scores were used for analyses. Codes are briefly described below and published in full at https://osf.io/dxe73/files/56csp.

### 2.5 Data Analyses

We first examined group differences and correlations between variables using R (Ben‐Shachar et al. 2020; Harrell and Dupont 2022; R Core Team 2023; Wickham et al. 2020). We then used multilevel modeling and actor‐partner interdependence modeling (APIM; Cook and Kenny 2005) strategies using HLM 8.0 software (Raudenbush et al. 2019). APIM modeling allowed us to test for actor effects (where one person's predictor variable predicts their own outcome variable) and partner effects (where one person's predictor variable predicts their partner's outcome variable) of narrative themes on relationship satisfaction, allowing us to fully utilize the dyadic nature of our data and statistically account for interdependent and intercorrelated data within a couple. Specifically, as recommended by Kenny et al. (2006), a series of strategies was implemented to address the interdependence inherent in our dyadic data. First, all four paths (two actor and two partner) were included in all analyses. Second, correlations between husbands' and wives' data were estimated in all analyses. This task was accomplished by including both husbands' and wives' predictors in each equation. Third, error terms (residuals) for husbands and wives were included in all analyses. The fourth strategy was to predict husbands' versus wives' residuals when the variance in the baseline models was heterogeneous. Due to limited power, residuals were not estimated as outcome variables, though husband and wife residuals were included as predictors in all analyses.

At Level 1, we included relationship satisfaction as the outcome (*Y*) and entered dummy codes for husbands (β_1_, coded 1 for husbands and 0 for wives) and wives (β_2_, coded 1 for wives and 0 for husbands) to set up a double intercept model, as well as an error term (*r*). At level 2, for each parameter (β_1_, β_2_) we included an intercept (γ_10_), followed by one set of predictors for husband narrative codes (γ_11_) and wife narrative codes (γ_12_), with error terms constrained for both parameters, to determine the associations between each partner's narrative codes and relationship satisfaction (including within‐person and actor‐partner associations). As an example, within the level 1 model—*Y* (relationship satisfaction) = β_1_ (husband dummy code) + β_2_ (wife dummy code) + *r* (error) – to estimate the effects of affective themes on husband relationship satisfaction, we modeled in level 2 the equation, β_1_ (husband relationship satisfaction) = γ_10_ (intercept) + γ_11_ (husband affective themes) + γ_12_ (wife affective themes).

All couples were assigned a couple identification number (in addition to an individual participant identification number), which matched their data across level 1 and level 2 data files. As is typical in narrative identity research (e.g., Adler et al. 2017), we ran analyses separately for each narrative predictor.

## 3 Results

### 3.1 Descriptive Analyses

The narratives were 153.03 words long on average (SD = 127.36). They described a total of 164 challenges across 13 categories including limited access to activities and movement (21.34%), adjusting to more of life's activities happening at home (14.02%), fears around contracting COVID‐19 (12.20%), work stressors (11.59%), marital conflict (10.98%), finances (6.71%), parenting/caregiving (6.10%), and mental health (4.88%). Fewer than 3% of participants stated there was no challenge to report or described challenges related to non‐immediate family (i.e., in‐laws or family of origin), physical health, resource scarcity, loss of loved ones (death), or concerns about the community or national response to COVID‐19.

The full range of each narrative code was represented in the data. Husbands' and wives' scores on narrative codes did not significantly differ from each other, with one exception: wives' scores (*M* = 2.34, SD = 0.76) on meaning‐making were higher on average than husbands' scores (*M* = 1.95, SD = 0.98), *Z* = −2.28, *p* = 0.02, *r*_rb_ = 0.23. Within‐spouse correlations across codes were generally small for husbands and ranged from small to moderate for wives, with three exceptions in which correlations were high.

For relationship satisfaction, the mean score was 65.20 (SD = 13.59) across the sample, placing it above the clinical cutoff (51.5) for relational distress. Thus, on average, spouses in this sample scored in the satisfied range. These findings are consistent with published studies of other newly married samples and of other samples recruited via the same method (e.g., Brock and Lawrence 2008; Lawrence and Bradbury 2007). There was also significant variability in spouses' satisfaction scores, which ranged from 15 to 81. Husbands' and wives' mean satisfaction scores did not significantly differ from each other. Please see Table S1 for full descriptives and bivariate correlations.

### 3.2 Which Narrative Themes Were Associated With Relationship Satisfaction?

We coded a suite of well‐established narrative themes (affect, agency, communion, and meaning‐making) and new relational narrative themes developed for this study (challenge outcome, couple stress response, familismo, and overriding positive regard) to clarify the extent to which narrative themes would associate with one's own and one's partner's relationship satisfaction. We first examined the simple relationships between themes and relationship satisfaction. As shown in Table 1, husbands' affect, agency, and communion scores were significantly correlated with their own relationship satisfaction scores, and wives' affect, challenge outcome, communion, couple stress response, familismo, and overriding positive regard were correlated with their own relationship satisfaction scores.

**TABLE 1:** Associations between narrative themes and relationship satisfaction by spouse.

Narrative theme	Husbands' CSI‐16		Wives' CSI‐16	
	*r*	*p*	*r*	*p*
Affect	**0.29**	**0.027**	**0.48**	**< 0.001**
Agency	**0.30**	**0.023**	0.13	0.328
Challenge outcome	0.10	0.461	**0.52**	**< 0.001**
Communion	**0.40**	**0.002**	**0.56**	**< 0.001**
Couple stress response	0.19	0.144	**0.46**	**< 0.001**
Familismo	0.17	0.207	**0.29**	**0.027**
Meaning‐making	−0.17	0.212	0.09	0.496
Overriding positive regard	−0.06	0.680	**0.39**	**0.002**

*Note:* Significant values are bolded.

**FIGURE 1:**
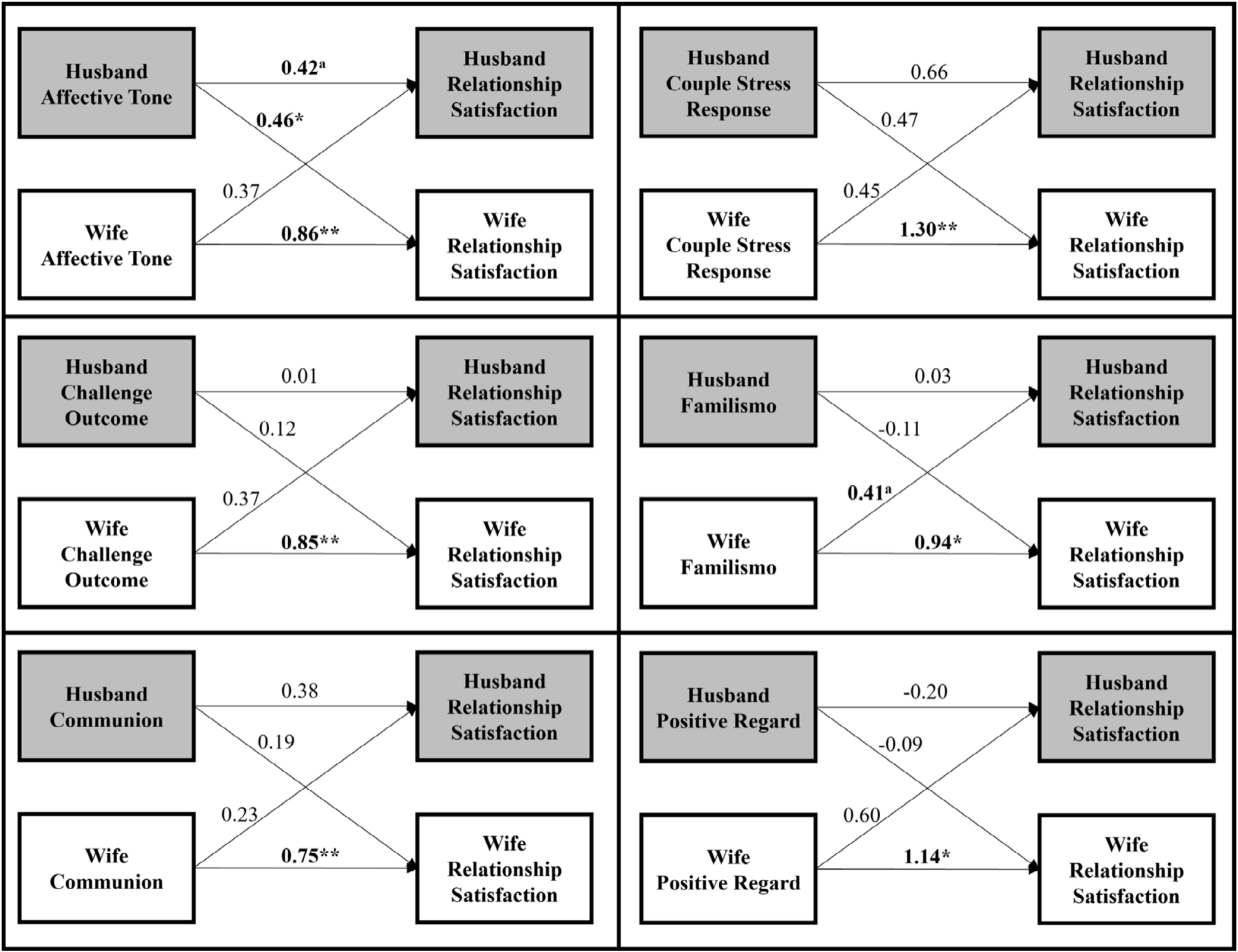
Results of APIM multilevel models of narrative themes predicting relationship satisfaction. Models using narrative themes of agency and meaning‐making are not displayed due to the lack of significant findings. ^a^ *p* < 0.10, *p < 0.05, **p < 0.01.

The results of our APIM analyses are shown in Table 2 and Figure 1. Most of the findings depict actor effects for wives. Specifically, wives had higher levels of relationship satisfaction when their narratives included stronger themes of positive affect and positive challenge outcomes, with medium effect sizes (*r*_effect_ = 0.30–0.32). Small effect sizes (*r*_effect_ = 0.19–0.28) were found for associations between wives' relationship satisfaction and their themes of communion, positive couple stress response, familismo, and overriding positive regard. We found one small partner effect (*r*_effect_ = 0.22): husbands with stronger narrative themes of positive affect had wives with higher relationship satisfaction.

**TABLE 2:** APIM models of narrative themes predicting relationship satisfaction.

	Parameter	Affect	Agency	Chall. out.	Communion	Cpl stress	Familismo	Meaning	Pos. regard
*Husband satisfaction*									
Intercept	Coefficient	4.86***	5.17***	5.21***	4.85***	5.40***	4.88***	5.66***	5.13***
	SE	0.42	0.25	0.37	0.39	0.21	0.36	0.76	0.57
	*r*_effect_	0.74	0.89	0.80	0.76	0.92	0.79	0.58	0.65
Husband theme	Coefficient	**0.42** ^**a**^	0.35	0.01	0.38	0.66	0.03	**−0.37** ^**a**^	−0.20
	SE	0.23	0.26	0.34	0.25	0.49	0.45	0.20	0.41
	*r*_effect_	0.17	0.13	0.00	0.15	0.13	0.01	0.17	0.05
Wife theme	Coefficient	0.37	0.22	0.37	0.23	0.45	**0.41** ^**a**^	0.20	0.60
	SE	0.27	0.19	0.28	0.28	0.48	0.48	0.29	0.51
	*r*_effect_	0.13	0.11	0.12	0.08	0.09	0.18	0.06	0.11
*Wife satisfaction*									
Intercept	Coefficient	4.54***	5.25***	4.97***	4.64**	5.44***	5.01***	4.81***	4.78***
	SE	0.36	0.28	0.30	0.34	0.20	0.38	0.79	0.58
	*r*_effect_	0.77	0.87	0.84	0.79	0.94	0.78	0.50	0.62
Husband theme	Coefficient	**0.46** *	0.37	0.12	0.19	0.47	−0.11	0.05	−0.09
	SE	0.20	0.28	0.26	0.21	0.38	0.42	0.19	0.41
	*r*_effect_	0.22	0.13	0.04	0.09	0.12	0.02	0.02	0.02
Wife theme	Coefficient	**0.86** **	0.15	**0.85** **	**0.75** **	**1.30** **	**0.94** *	0.25	**1.14** *
	SE	0.24	0.24	0.26	0.22	0.42	0.46	0.31	0.50
	*r*_effect_	0.32	0.06	0.30	0.31	0.28	0.19	0.08	0.21

Abbreviations: Chall. out., challenge outcome; cpl stress, couple stress response; meaning, meaning‐making; pos. regard, overriding positive regard; satisfaction = CSI‐16.

^a^ *p* < 0.10.

* *p* < 0.05.

** *p* < 0.01.

*** *p* < 0.001.

Though we use the standard significance level of *p* < 0.05 to evaluate our findings, we note three marginally significant trends of narrative themes that predicted husbands' relationship satisfaction. Specifically, husbands trended toward higher relationship satisfaction when their narratives had stronger themes of positive affect (*p* = 0.070) and weaker themes of meaning‐making (*p* = 0.071). Additionally, there was a trend toward a partner effect, with husbands reporting higher relationship satisfaction when their wives' narratives featured themes of familismo (*p* = 0.054). Effect sizes were small in each case (*r*_effect_ = 0.17–0.18), and we exercise caution in interpreting these findings.

To illustrate the nature of these themes in spouses' narratives, we first offer a brief narrative of a wife who scored three‐quarters of a standard deviation below the sample's average on relationship satisfaction:

The conditions of the pandemic…have made me feel extremely dissatisfied with our lifestyle. We've been constantly consuming screen time, but not really connecting with each other….We've [tried to deal with this problem by talking] about it on several occasions but not really come to any sort of agreement of what kind of life we would like to build together. We don't really have any mutual hobbies except entertaining/hosting which hasn't been allowed by the stay‐at‐home orders. It seems like the things that I like to do… he does not enjoy and the things that he likes to do… I do not enjoy. We've never really figured out the compromise dance. We kind of just take turns getting our way, but sometimes I feel like he gets his way more often…

I think the underlying issue is a sense of inequality in our relationship. I put some of my dreams and desires on hold when we got married and feel like I've sacrificed a lot for him to have his dream job and the lifestyle and creature comforts he wants.… Sacrificial giving is a good thing in marriage, but I think I may be enabling some of the behaviors [and] that make[s] me feel like a martyr. My disappointments also reveal where I had high expectations for our relationship that were probably unrealistic. [And] our values and lifestyle expectations are not as aligned as I thought they were….

This participant exhibits low affect (e.g., disappointments and “extreme dissatisfaction”), particularly in her descriptions of unmet expectations and “not really connecting with each other” (low communion). She observes that the challenges of coping with the pandemic as a couple have exacerbated pre‐existing problems (low couple stress response), and she engages in high meaning‐making in determining that their “values and lifestyle expectations are not as aligned as [she] thought.” She reaches this conclusion by recognizing that she put her own “dreams and desires on hold when we got married,” which led her to “feel like a martyr,” despite her belief that “sacrificial giving is a good thing in marriage” (an idea related to familismo).

Her husband also speaks to the general challenge of having limited options for activities due to lockdown. He describes their challenge as experiencing:

Restlessness and being stuck inside for prolonged periods of time. The news and media is [*sic*] frustrating. It's hard not knowing what's going to happen…I don't think there is a meaning or purpose for all of this. I'm quite skeptical that things will go back to normal anytime soon or be better. I still love my wife and want to make her happy and laugh so I try not to expose her to as much as I see.

This participant scored just over one standard deviation above the mean relationship satisfaction score of the sample, which is consistent with his closing statement demonstrating positive communion: “I still love my wife and want to make her happy and laugh.” Despite his worries about the broader global challenge of the pandemic, his narrative lacks the intensity of affect or the meaning‐making of his spouse's narrative. He does not draw deeper meanings about the relationship's quality in relation to the couple's challenge (zero score for couple stress response), nor does he directly connect the challenge to the global positive qualities of his relationship or his wife (absent positive regard).

## 4 Discussion

The purpose of the present study was to clarify the narrative themes that emerge in Mexican American couples' stories of relationship challenges at the beginning of the COVID‐19 pandemic and to examine how those themes relate to their relationship satisfaction. We found overlapping but distinct associations between narrative themes about COVID‐19 challenges and relationship satisfaction across husbands and wives, and we saw a more general pattern that wives' themes (but not husbands') tended to significantly predict their own satisfaction. This study adds to a nascent but growing body of narrative identity studies that use dyadic data analyses to study couples, including studies of how partners' stories of themselves and their partners may have similar themes (Panattoni and Thomsen 2018), the effects of attachment and narrative themes of relationship stories on both partners' relationship quality (Dunlop et al. 2020), and how both partners' relationship memories reflect satisfaction of relationship needs and predict both partners' relationship quality (Philippe et al. 2013).

Specifically, our findings offer evidence that narrative themes tend to be linked to self‐reported relationship satisfaction for both women and men, though there were fewer significant effects for men. Our APIM models, however, showed actor effects only for women and only one partner effect across all models. The gender asymmetry in our findings is consistent with a body of research suggesting that women tend to take on the “emotional labor” of relationships more than men, meaning they actively attend to their partners' emotions and work to generate more positive emotions (e.g., Horne and Johnson 2019). Our findings also add to a small group of previously published studies of Mexican American couples that show varied gender differences in relationship outcomes and associated variables (Capistrant et al. 2020; Negy and Snyder 1997; Peek et al. 2006).

### 4.1 Narrative Affect, Meaning‐Making, Agency, and Communion

Our study found that positive affect was associated with relationship satisfaction for both husbands and wives, consistent with prior narrative identity literature (Dunlop et al. 2020; Frost 2012) as well as marital research showing that expressions of affect are predictive of relationship satisfaction, concurrently and longitudinally (Mattson et al. 2011). This finding might also be interpreted as aligned with research on the Mexican American cultural value of *simpatía*, which promotes the outward expression of positive affect as a means of maintaining interpersonal harmony, even in situations of significant adversity (Martins et al. 2025). We did not make any hypotheses related to meaning‐making, nor did we find that it was associated with relationship satisfaction. However, we note that women scored higher than men on this construct, which may be consistent with some research suggesting women are more likely than men to integrate negative events into their sense of identity (Boals 2010).

In addition, our study is the first we are aware of in the narrative identity literature to demonstrate that higher scores on narrative themes of agency (for husbands) and communion (for both husbands and wives) are associated with greater relationship satisfaction. The latter finding is conceptually aligned with previous narrative identity research showing communion in relationship stories is associated with higher self‐reported relationship functioning (Dunlop et al. 2019), and with findings showing that greater emotional intimacy (Laurenceau et al. 2005) and communion (Ta 2017) are associated with higher relationship satisfaction. In addition, our finding that agentic stories were associated with higher relationship satisfaction for men, but not women, may suggest that men's feelings about their relationships may be particularly sensitive to their perception of the degree of agency or control they individually or as a couple exert to overcome challenges (Holmberg et al. 2003), which could be linked to previously reported associations between masculinity and problem‐solving (Wang 2007). This gender difference merits further investigation, particularly in the cultural context of this sample. Although much research (though not the present research) has demonstrated that men score higher in agency than women (Hsu et al. 2021), further research exploring connections among agency, communion, *machismo, marianismo*, and relationship outcomes may allow for greater nuance in interpreting these findings.

Nonetheless, together these findings suggest that several narrative themes associated with better individual psychological well‐being in the literature (Adler et al. 2015) are also linked to higher‐quality romantic relationships. These conceptual links suggest that narratives about couple experiences of navigating challenges together may tap into characteristics of romantic relationships, including how partners feel about their relationship and how they see their relationship as adapting and evolving in response to external challenges.

### 4.2 Narrative Positive Regard, Challenge Outcome, Couple Stress Response, and Familismo

Our findings on couple‐focused codes align with previous literature in relationship science and extend this research to the narration of couple challenges during a major life event like the COVID‐19 pandemic. First, we found that wives who linked challenges during the COVID‐19 pandemic to overriding positive regard for partners, and whose narratives reflected a sense that the relationship had improved in some way during the pandemic (couple stress response), had higher relationship satisfaction (Hawkins et al. 2002; Murray and Holmes 1999). We also found that wives who described a greater sense of resolution to pandemic challenges (challenge outcome) had higher relationship satisfaction. These findings are consistent with literature showing that the ability to cope effectively (or not) with shared external stress has the capacity to lead to relationship enhancement (or deterioration; Falconier et al. 2015). These narrative themes may be linked to an underlying sense that one's relationship has a strong foundation, that partners love and like each other even when life circumstances are difficult, that couples can overcome difficulties together, and/or that relationships can grow in the face of stress. Additionally, these qualities of relationship stories may create positive feedback loops that contribute to better relationship experiences during times of stress.

Lastly, familismo, reflecting a deep sense of connectedness and commitment to one's family and prioritization of overall family closeness, cohesion, and positivity, was also associated with relationship satisfaction for women. Familismo is a cultural value often identified as a source of strength in the literature (Cahill et al. 2021; Smith‐Morris et al. 2013), and we found that it was indeed highly associated with relationship satisfaction, representing a relationship strength among Mexican American couples. Possible mechanisms for this finding include a greater sense of closeness to family in general (including the spouse) or a tendency among Mexican‐origin couples relative to other ethnic groups to discuss problems more openly (Rodriguez et al. 2016).

### 4.3 The Benefits of Using a Narrative Identity Approach to Enhance Relationship Science

The narrative identity approach offers several advantages for exploring couples' experiences of relationship challenges and their associations with relationship satisfaction during periods of severe stress, and perhaps in other important life experiences. First, eliciting a single, personal story—even one about difficult events—is pleasant for most participants (Turner et al. 2024), is relatively brief, and generates rich data that can be utilized to answer multiple questions. In our study, spouses were prompted to recall any highly salient COVID‐19–related marital stressor of their choosing, increasing the likelihood that they called upon a memory relevant to their identity as a partner and to their satisfaction with their marriage. Open‐ended prompting for challenges also allowed us to contribute to the literature about major couple challenges encountered during the COVID‐19 pandemic by drawing on individuals' lived experiences rather than relying on more general or stereotyped imagery.

Second, in bringing about these specific accounts, we asked partners to articulate autobiographical memories. Autobiographical memories are self‐memories created within layers of development, culture, and personality (Fivush 2010; Wang 2021) that, when expressed, illustrate how the inner world shapes interpretations of external events (Bluck 2003). Such accounts are reciprocally related stories that relay how individuals remember the past and interpret the present. Because interpretations of the past may affect behaviors and cognitions in the present (Wilson and Ross 2003), examining these narratives may offer insights into the present not easily accessed otherwise. Third, narrative identity methods allow us to quantify qualitative data such that individuals' idiosyncratic approaches to narrating challenging experiences can be linked to objective outcome measures. Extending this, the unique dyadic nature of our data offers the opportunity not only to investigate how individuals' narrative identities are linked to their own relationship satisfaction, but also to how individuals' narrative identities are linked to those of their partners.

### 4.4 Strengths and Limitations

Several methodological strengths of the present study enhance our confidence in our results. First, we collected data from both partners, yielding narratives from husbands and wives talking about the same relationship in the context of the same broad, global stressor. This also allowed us to account for interdependence among dyads and examine actor and partner paths statistically. Second, we collected data in real time during the lockdown rather than retrospectively, which enhances our confidence that participants were truly having these thoughts at the time. Third, we enhanced internal reliability by using a sample that was homogeneous in terms of first marriages and length of marriage. Fourth, we examined Mexican American dyads, an important population given their increased risk for negative outcomes from the pandemic, their increasing proportion of the US population, and the relative dearth of knowledge about Mexican American couples (compared to non‐Hispanic White couples). We examined Mexican American couples specifically, as different communities within a given ethnicity will differ and should be studied separately. Fifth, to better capture the full range of Mexican American couples, we included couples in which one or both partners were Mexican American, rather than limiting our sample to only same‐ethnicity couples.

Additionally, several factors may limit the generalizability of our findings. First, all measures were administered in English, and participants were required to be sufficiently proficient in reading and writing in English to participate. The findings should be replicated with a Mexican American sample in Spanish. Second, the data are cross‐sectional and, as such, temporal precedence between narrative themes and relationship satisfaction cannot be ascertained. Because our sample consisted wholly of newly married community couples, the findings may not generalize to distressed treatment‐seeking couples, more “established” relationships (i.e., couples married for more than 5 years), or couples recruited via other methods. For example, established couples may exhibit characteristic differences in how they narrate relationship stories (Wilson and Kiecolt‐Glaser 2022). Finally, though our decision not to correct for multiple statistical tests aligns with other narrative identity studies, it increases the potential for Type I errors in our results and underscores the need for future research with larger samples.

### 4.5 Implications of the Present Study

Our study was prompted and shaped by the COVID‐19 pandemic and the unprecedented stresses it placed on couples and Mexican American families; as such, the findings are inextricably linked to this context. At the same time, our methods, data, and findings raise several interesting questions for future research that extend beyond COVID‐19 and this population. First, it would be important to replicate this research approach with other groups of Mexican American individuals and to extend it to individuals of sexual and gender minorities, as well as to other Hispanic groups and other racial and ethnic identities. We expect that trained coders could apply the narrative themes investigated in this study to predict relationship satisfaction in relationship challenge narratives of early to midlife adults in the United States. Second, our narratives were collected early in the COVID‐19 pandemic. It would be illuminating to collect similar data on other major global or personal challenges and to investigate whether our findings are unique to the pandemic context or extend to other life‐changing circumstances in couples' lives. Third, given the novelty of some of our codes and findings, these quantified narrative theme codes and their associations with relationship satisfaction and other relationship measures must continue to be tested and refined. Finally, an examination of how actor‐partner effects may differ when analyzing relationship challenge stories narrated by individuals versus those that are co‐narrated (Koenig Kellas et al. 2010) or told vicariously (Panattoni and Thomsen 2018) may help clarify the mixed results across these data collection methods.

The current dataset is rich in qualitative data that might be analyzed using different means. For example, future studies might build on previous research using a grounded theory approach to identifying relevant narrative themes related specifically to relationship functioning. We also draw attention to the mutually constitutive nature of gender and narrative, both of which involve meaning‐making processes shaped by cultural master narratives (Fivush and Grysman 2022), including those that exclude minoritized and marginalized individuals (Syed and McLean 2022). The potential to deeply examine the complex relationships among gender, ethnicity, meaning‐making, and relationship functioning through stories of lived experience is particularly promising. As researchers work to expand representation in couples research, it is crucial to include diverse methodologies, such as narrative identity methods, that broaden perspectives beyond those accessible through self‐report measures (Righetti et al. 2022; Totenhagen et al. 2023).

Clinically, the findings from this study add to the growing body of evidence that culturally specific concepts should be brought into the couple therapy room. For example, greater acculturative stress is associated with worse couple outcomes (Gonzalez‐Detrés et al. 2025). Mexican American couples faced significant increases in acculturative stress starting in 2016 (when the present sample was recruited) due to political and national policy changes. Acculturative stress then increased during the pandemic due to the unique stressors facing Latine couples during that time. Given the findings from the present study and others showing that familismo offers various protective benefits for couples as they cope with major stressors such as acculturative stress (Boron et al. 2024; Rodriguez et al. 2016), the way familismo (and other culturally specific concepts such as simpatía, machismo, and marianismo) is storied by partners might be directly targeted within couple therapy.

Understanding how the couples in our study navigated the pandemic can offer valuable insights into the resilience of the fastest‐growing group in the United States, their coping mechanisms, and the influence of cultural values on family dynamics. Examining their experiences also sheds light on how external stressors, such as public health crises, affect romantic partners and family relationships, particularly in communities that experience disparities. This research may contribute to a more nuanced understanding of Mexican American couples in the United States, guiding policy development and interventions that support their specific needs and foster stronger communities and healthier family relationships.

## Conflicts of Interest

The authors declare no conflicts of interest.

## Supporting information


**Table S1:** Descriptives and correlations among narrative themes and relationship satisfaction.

## Data Availability

The quantitative data and materials that support the findings of this study are openly available in https://osf.io/dxe73. Qualitative data are not publicly available due to participant privacy concerns.

## Endnotes

^1^
In the literature review, we have used the terms used in each publication (e.g., Hispanic, Latinx). Otherwise, we use Mexican American to refer to the present study sample.
