# Supplementary material for: Narratives About COVID Challenges in Mexican American Couples as Predictors of Relationship Satisfaction
Source: Fam Process. 2026 Jul 2;65(3):e70176. doi: 10.1111/famp.70176 (PMC13329209; doi:10.1111/famp.70176)
Supplement: Supplementary file 1 — Table S1: Descriptives and correlations among narrative themes and relationship satisfaction. [file FAMP-65-0-s001.pdf]

Narratives about COVID challenges in Mexican American couples as predictors of relationship satisfaction

Hollen N. Reischer, Hayley C. Fivecoat, Lena Blum, Chrishane N. Cunningham, Stacey Ho, and Erika Lawrence

Supplemental Materials

Table S1

Descriptives and Correlations Among Narrative Themes and Relationship Satisfaction

Variable	Affect	Agency	Chall. out.	Communion	Cpl stress	Familismo	Meaning	Pos. regard	CSI-16	Husbands *M* (*SD*) or % present
Wives					Husbands					
1. Affect	.00	.32**	.33**	.65***	.40**	.43**	.24*	.49***	.29*	0.66 (0.97)
2. Agency	.46***	.30*	.50***	.47***	.18[a]	.20[a]	.35**	.17	.30*	0.52 (1.00)[a]
3. Chall. out.	.75***	.27*	.20	.51***	.20*	.05	.42***	.47***	.10	0.53 (0.71)
4. Communion	.80***	.38**	.75***	.43***	.36**	.26[a]	.30*	.44***	.40**	0.95 (1.00)
5. Cpl stress	.58***	.36**	.60***	.66***	.08	.27*	.00	-.04	.19	-0.02 (0.44)
6. Familismo	.27*	.06	.41**	.26[a]	.06	.50***	.15	.29*	.17	44.82%
7. Meaning	.15	.12	.23[a]	.11	-.03	.18	.05	.41***	-.17	1.95 (0.98)*
8. Pos. regard	.71***	.27*	.59***	.73***	.46***	.37**	.33**	.00	-.06	53.45%
9. CSI-16	.48***	.20	.52***	.56***	.46***	.29*	.09	.39*	.59***	65.14 (14.02)
Wives *M* (*SD*) or % present	0.76 (0.88)	0.26 (0.97)	0.52 (0.94)	0.86 (1.02)	0.03 (0.53)	55.17%	2.34 (0.76)	65.52%	65.47 (14.33)	

Note. Chall. out. = challenge outcome; cpl stress = couple stress response; meaning = meaning-making; pos. regard = overriding positive regard; CSI-16 = relationship satisfaction. Correlation coefficients for husbands ($n = 58$) are above the diagonal in blue, and for wives ($n = 58$) below the diagonal in purple. Correlations along the diagonal in white represent cross-spouse correlations within each measure ($n = 58$). In the final row and column, means and standard deviations are presented for all ordinal variables, and percent of "present" scores are displayed for binary variables. Correlation

coefficients are Pearson's *r* for continuous variables, point-biserial correlation coefficients for the associations between binary variables and continuous variables, and phi coefficients derived from chi-square tests when both variables were binary.

[a]$p < .10$, *$p < .05$, ** $p < .01$, ***$p < .001$
